# The St. George’s Respiratory Questionnaire as a prognostic factor in IPF

**DOI:** 10.1186/s12931-017-0503-3

**Published:** 2017-01-17

**Authors:** Taiki Furukawa, Hiroyuki Taniguchi, Masahiko Ando, Yasuhiro Kondoh, Kensuke Kataoka, Osamu Nishiyama, Takeshi Johkoh, Junya Fukuoka, Koji Sakamoto, Yoshinori Hasegawa

**Affiliations:** 1 Department of Respiratory Medicine and Allergy, Tosei General Hospital, 160 Nishioiwake-cho, Seto, Aichi 489-8642 Japan; 2 Center for Advanced Medicine and Clinical Research, Nagoya University Hospital, 65 Tsurumai-cho, Showa-ku, Nagoya, Aichi 466-8650 Japan; 3 Department of Respiratory Medicine and Allergology, Kindai University, Faculty of Medicine, 377-2 Onohigashi, Osaka-sayama, Osaka 589-8511 Japan; 4 Department of Radiology, Kinki Central Hospital of Mutual Aid Association of Public Health Teachers, 3-1 Kurumazuka, Itami, Hyougo 664-8533 Japan; 5 Department of Pathology, Nagasaki University Graduate School of Biomedical Sciences, 1-7-1 Sakamoto, Nagasaki, Nagasaki 852-8501 Japan; 6 Department of Respiratory Medicine, Nagoya University Graduate School of Medicine, 65 Tsurumai-cho, Showa-ku, Nagoya, Aichi 466-8550 Japan

**Keywords:** Health related QoL, Idiopathic pulmonary fibrosis, Prognostic factors, The St. George’s Respiratory Questionnaire

## Abstract

**Background:**

It is unclear whether health related quality of life (HRQL) may have a predictive value for mortality in idiopathic pulmonary fibrosis (IPF).

We investigated the relationship between HRQL assessed using the St. George’s Respiratory Questionnaire (SGRQ) and survival time in patients with IPF, and tried to determine a clinical meaningful cut off value to predict poorer survival rates.

**Methods:**

We retrospectively analyzed consecutive patients with IPF who underwent an initial evaluation from May 2007 to December 2012. The diagnosis of IPF was made according to the 2011 international consensus guidelines. We used Cox proportional hazard models to identify independent predictors for mortality rate in patients with IPF.

**Results:**

We examined 182 eligible cases, average age was 66 years old, and 86% were male. Mean levels of percent predicted FVC, DLco, six-minute-walk test distance, and the SGRQ total score were around 80%, 58%, 580 m, and 34 points. On multivariate analysis, the SGRQ total score (hazard ratio [HR], 1.012; 95% confidence interval [CI] 1.001–1.023; *P* = .029) and percent predicted FVC (HR, 0.957; 95% CI 0.944–0.971, *P* < .001) were independent predictors for mortality rate. Moreover, a score higher than 30 points in the SGRQ total score showed higher mortality rate (HR, 2.047; 95% CI, 1.329–3.153; *P* = .001).

**Conclusions:**

The SGRQ total score was one of independent prognostic factors in patients with IPF. Total scores higher than 30 points were associated with higher mortality rates.

**Trial registration:**

This study was retrospective, observational study, so it is not applicable.

**Electronic supplementary material:**

The online version of this article (doi:10.1186/s12931-017-0503-3) contains supplementary material, which is available to authorized users.

## Background

Idiopathic pulmonary fibrosis (IPF) is a fatal lung disease characterized by chronic, progressive fibrosing interstitial pneumonia of unknown etiology [1]. As the disease condition progresses, dyspnoea on exertion becomes severe and health-related quality of life (HRQL) seriously deteriorates [2].

HRQL is a subjective and multidimensional appraisal that focuses on the impact of illness and treatment on physical, emotional, and social well-being [3]. HRQL tools can thus capture various information that physiologic or radiologic measures cannot. Therefore, it has been considered to be one of the most important endpoints in pharmacological trials and rehabilitation for IPF [4, 5].

The St. George’s Respiratory Questionnaire (SGRQ) is the most frequently used tool to measure HRQL. It was developed as a standardized, self-administered, disease-specific health status evaluation scale for patients with chronic obstructive disease [6]. The questionnaire consists of 50 items with 76 weighted responses that produce scores in three domains and one total score. The domains are symptoms (breathlessness, cough, and wheeze), activities (that are limited by the symptoms), and impacts (social and psychologic effect of the respiratory diseases). The score for each domain is calculated from 0 to 100, with higher scores corresponding to worse HRQL.

In patients with COPD, the SGRQ was shown to be a significant predictor of mortality [7, 8]. However, in patients with IPF, the relationship between HRQL assessed using the SGRQ and mortality has not fully been studied.

The aim of the present study was to investigate whether the SGRQ has a predictive value for mortality in IPF, including various dimensional clinical factors previously reported as prognostic factors, namely MMRC [9], % predicted FVC, % predicted DLco [1, 10–15], 6MWD, desaturation during 6MWT [13, 16–18], and BMI [19]. We also sought a clinically meaningful cut off value to predict poorer mortality rate.

## Methods

### Study subjects

Two-hundred and five consecutive patients with IPF, who underwent systemic initial evaluation from May 2007 to December 2012 at a center for respiratory diseases (Aichi, Japan), were identified from their medical records. The diagnosis of IPF was made according to the 2011 international consensus guidelines, and since 2011 the accuracy of the diagnosis has been confirmed by multidisciplinary discussion (MDD); however, some of the patients who initially presented before 2010 had been diagnosed based on different guidelines. The diagnosis for these patients was therefore confirmed according to the 2011 guidelines and MDD before May 2015 [1]. A thoracic radiologist with 27 years of experience re-read the chest high-resolution CT (HRCT) that had been obtained at the initial evaluation. This thoracic radiologist was blind to the clinical course and examination data for each patient.

Patients were excluded from the present study if there was clinical evidence of other known conditions, such as connective tissue disease, left heart failure, an occupational or environmental exposure that may result in interstitial lung disease, or a history of ingestion of an agent known to cause interstitial lung disease. Moreover, patients who had been prescribed medication for IPF (i.e. anti-fibrotic drug, corticosteroid, immunosuppressant), or who had undergone long-term oxygen therapy before initial evaluation at a center for respiratory diseases (Aichi, Japan) were also excluded. Finally, we examined 182 patients with IPF who were newly diagnosed. Of them, 55 patients who were in a clinical trial were enrolled. The present study was approved by a local institutional review board (IRB No. 509).

### Data collection

Clinical data were retrospectively collected from a medical chart review. The eligible patients had undergone all of the tests and assessments that were physical examination and assessment of physiological function, dyspnoea (modified Medical Research Council (MMRC) scale and Baseline Dyspnoea Index (BDI) score), exercise capacity (6-minute-walk test (6MWT) and desaturation during 6MWT), and HRQL, which was assessed using the SGRQ [6] at the initial evaluation. The Japanese version of the SGRQ has been previously validated [20].

All patients completed pulmonary function tests (PFTs) by spirometry (CHESTAC-55 V; Chest, Tokyo, Japan), according to the ATS/ERS criteria [21]. Diffusion capacity for carbon monoxide (DLco) was also measured (CHESTAC-55 V). The values for forced vital capacity (FVC), forced expiratory volume in 1 second (FEV1), and DLco were measured according to the American Thoracic Society/European Respiratory Society recommendation [22]. The 6MWT was performed according to the ATS/ERS criteria [23]. The duration from initial evaluation to the last attendance or death was recorded. We analyzed censored cases by calling them to confirm their life-or-death status.

### Statistical analysis

We performed all analyses by using SPSS (version 22; Chicago, IL, USA). Clinical variables were used as continuous variables, except that the categorical variables of gender, smoking status, and a history of surgical lung biopsy were coded as one or zero for the analyses. Continuous variables were presented as mean ± standard deviation unless otherwise stated. Categorical variables were reported as counts and percentages. The survival time was calculated with the life table method. We performed univariate and multivariate Cox proportional hazards analyses to investigate the relationships between clinical variables and mortality rate with adjustment for age and gender. Results of Cox proportional hazards analyses were presented in terms of estimated hazard ratios (HRs) with corresponding 95% confidence intervals (CI); *p* values of less than 0.05 were considered to be statistically significant. In order to select final prognostic predictors in multivariate analysis, all candidate predictors for which the *p*-value was < 0.1 on univariate analysis were included in a forward selection method, with a *p*-value of 0.05 used for final entry or removal. To avoid multicollinearity, some of the highly correlated variables were excluded on multivariate analysis if they had Pearson’s correlation coefficients higher than 0.7. We performed a receiver operating characteristic (ROC) curve analysis to find an optimized cutoff value for 3-year survival prediction. Survival curves were estimated using the Kaplan–Meier method, and compared using the log-rank test across the higher and lower groups of the SGRQ total score.

## Results

### Patient characteristics

The characteristics of the study population are shown in Table 1. Average age was 65.6 years old, and 85.2% of the patients were male. Surgical lung biopsy was performed to diagnose IPF in 97 patients (53.3%). Median follow-up time was 36.1 months (interquartile range, 19.3 to 48.6 months), 12 cases were lost to follow-up, and 94 patients (51.6%) died within the follow-up times. MST was 48.3 months (95% CI, 43.5 to 53.1 months).

**Table 1:** Baseline characteristics of study population^a^ (*N* = 182)

Characteristic	
Age, y	65.6 ± 8.0
Male gender, N (%)	155 (85.2)
BMI, kg/m^2^	24.0 ± 3.4
Ever smoking, N (%)	147 (81.9)
Surgical Lung Biopsy, N (%)	97 (53.3)
Arterial blood gas analysis	
PaO_2_, mmHg	82.0 ± 11.6
Pulmonary function	
FVC, % predicted	79.7 ± 19.1
FEV_1_ / FVC	85.2 ± 7.5
DLco, % predicted (*N* = 179)	58.2 ± 20.1
Dyspnoea index	
MMRC (0/1/2/3/4)	51 / 74 / 41 / 15 / 1
BDI (*N* = 180)	8.9 ± 2.4
6MWT (*N* = 181)	
6MWD, m	580 ± 136
SpO_2_ nadir, %	82.8 ± 9.1
SGRQ score	
Symptom	44.2 ± 22.6
Activity	40.0 ± 26.1
Impact	27.7 ± 19.8
Total	34.5 ± 20.2

*BMI* body mass index, *FVC % predicted* percent predicted forced vital capacity; *FEV*_*1*_ forced expiratory volume in the first second, *DL*_*CO*_ *% predicted* percent predicted diffusion capacity for carbon monoxide, *PaO*_*2*_ partial pressure for oxygen, *6MWD* 6-min walk distance, *SpO*_*2*_ oxygen saturation by pulse oximetry

^a^Plus–minus values are means ± SD

### Baseline levels of clinical indices

**Fig. 1:**
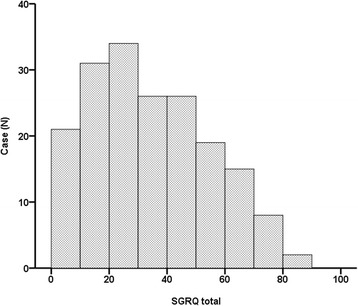
The distribution histogram for SGRQ

Table 1 shows all variables we collected in the present study: mean levels of PaO_2_, the pulmonary function test, exercise capacity, dyspnoea scale and HRQL score. Mean percent predicted FVC was 79.7%, mean percent predicted DLco was 58.2%. Mean score on the SGRQ total domain was 34.5 points and its distribution is shown in Fig. 1. The relationship between SGRQ and baseline physiological measures is shown in Additional file 1: Table S1. Comorbidities, especially diabetes and orthopedic disease, were related to SGRQ total score. However, they did not have predictive value for mortality with adjustment for age and gender (HR, 1.449; 95% CI 0.901–2.332, *P* = 0.13, HR, 0.748; 95% CI 0.325–1.720, *P* = 0.49, respectively) (Additional file 1: Table S2).

### Correlation between baseline levels of clinical indices and mortality

On univariate analysis, all domain scores of the SGRQ showed significant predictive value for mortality (Table 2). Moreover, symptom, impact, and total domain showed significant predictive value for mortality with adjustment for age, gender, and %FVC model (Additional file 1: Table S3). Because the SGRQ total score included all domain scores of the SGRQ and there were high correlations between the total score and each domain score, we only used the SGRQ total score in multivariate analysis. Moreover, BDI was excluded from multivariate analysis because of its high correlation with MMRC. In multivariate analysis using a forward selection method, the SGRQ total score was an independent predictor for mortality (HR, 1.012; 95% CI, 1.001–1.023; *P* = .029), along with percent predicted FVC (HR, 0.957; 95% CI, 0.944–0.971; *P*  <.001) (Table 3).

**Table 2:** Univariate Cox proportional-hazard analysis

Variables	Adjusted HRs (95% CI)^a^	*p* value
Age, y	1.005 (0.979–1.031)	0.72
Male gender	0.725 (0.394–1.334)	0.30
BMI, kg/m^2^	0.882 (0.826–0.942)	<0.001
PaO_2_, mmHg	0.978 (0.961–0.996)	0.014
FVC, % predicted	0.954 (0.941–0.967)	<0.001
DLco, % predicted	0.976 (0.964–0.989)	<0.001
MMRC	1.413 (1.14–1.753)	0.002
BDI	0.830 (0.764–0.903)	<0.001
6MWD, m	0.996 (0.994–0.998)	<0.001
SpO_2_ nadir, %	0.962 (0.944–0.98)	<0.001
SGRQ		
Symptom	1.019 (1.01–1.029)	<0.001
Activity	1.014 (1.006–1.022)	0.001
Impact	1.019 (1.009–1.029)	<0.001
Total	1.021 (1.011–1.032)	<0.001

^a^; age, gender adjusted

**Table 3:** Multivariate Cox proportional-hazard analysis

Variables	Adjusted HRs (95% CI)^a^	*p* value
FVC, % predicted	0.957 (0.944–0.971)	<0.001
SGRQ total	1.012 (1.001–1.023)	0.029

^a^; age, gender adjusted

**Fig. 2:**
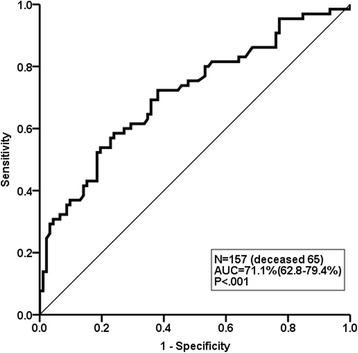
The ROC curve for SGRQ models in predicting 3-year mortality

**Fig. 3:**
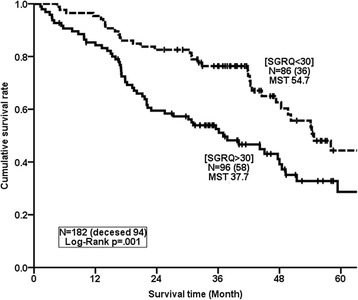
The Kaplan-Meier Curve for SGRQ models

The ROC curve analysis for the 3-year mortality rate demonstrated that the most accurate and optimal cutoff value for the SGRQ total score was 30 points (area under the curve, 71.1%; 95% CI 62.8–79.4%; *P* < .001) (Fig. 2). MST was significantly shorter in patients with SGRQ total scores of over 30 points than in patients with scores under 30 points (MST, 37.7 vs 54.7 months. HR, 2.047; 95% CI, 1.329–3.153; *P* = .001) (Fig. 3).

## Discussion

This is the first report to show that health status assessed using the SGRQ total score is an independent predictor of mortality in patients with IPF. Moreover, IPF patients with a SGRQ total score higher than 30 points had a higher mortality rate.

The SGRQ is widely used as an assessment tool for HRQL and is applied as one of the key endpoints in clinical trials in patients with IPF [24–29] and COPD [30–32]. However, the impact of HRQL (assessed using the SGRQ) on survival rate has not been fully studied in patients with IPF.

In the univariate analysis in this study, the SGRQ as well as various dimensional clinical factors previously reported as prognostic factors were significant predictors for mortality rate. However, the multivariate analyses showed that both the SGRQ as a patient-reported outcome and percent predicted FVC as a physiological indicator had superior capabilities as independent predictors of mortality than other widely known predictors. Baseline FVC has been reported to be a robust prognostic factor [12], and was recently included in a prognostic model [12]. The present study also shows that baseline FVC remains an important prognostic factor, and the SGRQ total score is an equally independent prognostic meaningful factor. This may be because the SGRQ is derived from multidimensional viewpoints of disease severity, and so may capture more comprehensive information than individual predictive variables.

However, there has been no study on an appropriate cut off value for the SGRQ total score in predicting mortality in patients with IPF. We showed that patients with SGRQ total scores higher than 30 points had a remarkably higher mortality, with a hazard ratio of 2.047. In contrast to the present results, SGRQ was not a significant predictive value for mortality in a previously reported multivariate analysis [33]. The authors of that study examined a smaller sample size and made diagnoses from 2000 to 2005 based on 2000 guidelines that were different from the 2011 ones with a formal multidisciplinary discussion (MDD) used in the present study.

The mean value of the SGRQ total score was reported by Swigris and colleagues to be around 45 points with 56.9–73.1% in FVC from a literature review [5], versus 34.5 points with 79.7% in FVC in the present study. Interestingly, the latest rigorous clinical trial of INPULSIS showed a SGRQ total score (39.6 points) and FVC (79.6%) closer to our findings. Considering the grave prognosis with a score higher than 30 points, these scores should be explicitly considered in the administration of anti-fibrotic drugs, even though FVC may be preserved.

Recently the importance of comorbidities in IPF was reported, however, the relationship between HRQL and comorbidity in IPF has not been fully examined. The present study showed that comorbidities, especially diabetes and orthopedic disease, were related to total score, however, they didn’t have predictive value in age and gender adjusted models.

The present study had some limitations. First, this is a retrospective, single-centered study, and so there may be selection bias. Further large prospective studies will be needed to assess the relationship between the SGRQ and mortality rate. Next, because we examined mild to moderate cases, results may differ in a different cohort. Lastly, all the patients in the present study were Japanese, and it is unclear whether the findings will apply to people of different ethnicities.

## Conclusion

The SGRQ total score was found to be an independent prognostic factor in patients with IPF, along with percent predicted FVC. Total scores higher than 30 points were associated with a higher mortality rate.

## Additional file

Additional file 1: Table S1. Spearman’s correlation coefficients between SGRQ and baseline physiological measures. **Table S2.** The relationship between SGRQ and comorbidities. **Table S3.** SGRQ domain as predictors for mortality. (DOCX 29 kb)

## Abbreviations

6MWD: 6-min walk distance
6MWT: 6-minute-walk test
BDI: Baseline Dyspnoea Index
CI: Confidence interval
DLco: Diffusion capacity for carbon monoxide
FEV1: Forced expiratory volume in 1 second
FVC: Forced vital capacity
HR: Hazard ratio
HRQL: Health-related quality of life
IPF: Idiopathic pulmonary fibrosis
MMRC: Modified Medical Research Council
MST: Median survival time
ROC: Receiver operating characteristic
SGRQ: St. George’s Respiratory Questionnaire
