# Supplementary material for: The St. George’s Respiratory Questionnaire as a prognostic factor in IPF
Source: Respir Res. 2017 Jan 17;18:18. doi: 10.1186/s12931-017-0503-3 (PMC5240376; doi:10.1186/s12931-017-0503-3)
Supplement: Additional file 1: Table S1. — Spearman’s correlation coefficients between SGRQ and baseline physiological measures. Table S2. The relationship between SGRQ and comorbidities. Table S3. SGRQ domain as predictors for mortality. (DOCX 29 kb) [file 12931_2017_503_MOESM1_ESM.docx]

| Table S1. Spearman’s correlation coefficients between SGRQ and baseline physiological measures. | | | | |
| --- | --- | --- | --- | --- |
| Characteristic | symptom | activity | impact | total |
| Age, y | 0.029 | 0.139 | 0.111 | 0.110 |
| BMI, kg/m^2^ | 0.017 | 0.086 | -0.052 | 0.017 |
| PaO_2_, mmHg | -0.183* | -0.290** | -0.175* | -0.237** |
| FVC, % predicted | -0.251** | -0.399** | -0.298** | -0.362** |
| FEV_1_ / FVC | 0.049 | 0.030 | 0.112 | 0.073 |
| DLco, % predicted (N=179) | -0.415** | -0.478** | -0.303** | -0.435** |
| MMRC | 0.518** | 0.710** | 0.573** | 0.683** |
| BDI (N=180) | -0.561** | -0.738** | -0.631** | -0.731** |
| 6MWT (N=181) |  |  |  |  |
| 6MWD, m | -0.353** | -0.601** | -0.407*: | -0.522** |
| SpO_2_ nadir, % | -0.352** | -0.329** | -0.278** | -0.341** |

*; p<0.05, **p<0.01

| Table S2. The relationship between SGRQ and co-morbidities | | | | | | | | | | | | | |
| --- | --- | --- | --- | --- | --- | --- | --- | --- | --- | --- | --- | --- | --- |
|  |  | Symptom | | | Activity | | | Impact | | | Total | | |
| Comorbidity | n | - | + | p value | - | + | p value | - | + | p value | - | + | p value |
| Heart failure (any cause) | 3 | 44.1±22.5 | 54.4±28.3 | 0.43 | 39.7±26.0 | 62.7±23.7 | 0.13 | 27.4±19.5 | 45.6±31.4 | 0.12 | 34.2±20.1 | 52.2±24.5 | 0.13 |
| Ischemic heart disease | 8 | 44.2±22.9 | 43.9±16.6 | 0.96 | 39.7±25.8 | 47.3±31.5 | 0.42 | 27.7±19.9 | 27.3±18.6 | 0.95 | 34.4±20.4 | 36.3±17.6 | 0.80 |
| Diabetes | 40 | 43.5±22.3 | 46.9±23.8 | 0.39 | 37.3±25.4 | 49.7±26.5 | 0.007 | 26.4±18.5 | 32.4±23.5 | 0.14 | 32.8±19.3 | 40.4±22.5 | 0.03 |
| Hypertension | 40 | 43.9±22.5 | 45.4±23.1 | 0.71 | 38.5±26.1 | 45.5±25.3 | 0.14 | 26.6±19.9 | 31.7±19 | 0.15 | 33.3±20.3 | 38.5±19.8 | 0.15 |
| Hyperlipemia | 28 | 44.9±22.4 | 40.3±23.8 | 0.32 | 39.3±26.2 | 44.2±25.4 | 0.36 | 28.0±20.2 | 26.3±17.5 | 0.68 | 34.6±20.5 | 33.9±18.9 | 0.87 |
| Orthopedic disease | 15 | 43.5±22.4 | 52.2±24.3 | 0.16 | 38.7±26.1 | 54.5±21.3 | 0.02 | 26.3±19.4 | 43.2±17.8 | 0.001 | 33.2±20.0 | 49.1±17.4 | 0.003 |
| Other respiratory disease | 10 | 43.1±22.3 | 63.7±18.9 | 0.005 | 39.6±26.1 | 47.4±26.3 | 0.36 | 27.0±19.7 | 40.4±17.4 | 0.04 | 33.8±20.2 | 46.5±16.8 | 0.053 |
| GERD | 3 | 43.9±22.6 | 63.2±6.0 | 0.14 | 39.7±26.1 | 60.9±16.1 | 0.16 | 27.4±19.7 | 43.9±20.2 | 0.15 | 34.2±20.2 | 52.3±14.9 | 0.13 |

| Table S3. SGRQ domain as predictors for mortality*. | | |
| --- | --- | --- |
|  | HR (95%CI) | p value |
| symptom | 1.014 (1.004-1.024) | 0.005 |
| activity | 1.005 (0.997-1.014) | 0.212 |
| impact | 1.010 (1.000-1.021) | 0.046 |
| total | 1.012 (1.001-1.023) | 0.029 |

*age, gender, and %FVC adjusted models
